# Bridging Gaps in Oral Health Frameworks: Mapping With Hodges' Health Career ‐ Care Domains ‐ Model

**DOI:** 10.1111/jphd.70034

**Published:** 2026-01-27

**Authors:** Silvana Bettiol, Peter Jones, Hyacinth A. Onyedikachi, W. George Kernohan

**Affiliations:** ^1^ Tasmania School of Medicine, College of Health and Medicine University of Tasmania Hobart Australia; ^2^ NHS Professionals and University of Greater Manchester Bolton UK; ^3^ Institute of Nursing and Health Research Ulster University Belfast UK

**Keywords:** conceptual frameworks, Hodges' health career domains model, integrated care, oral health

## Abstract

**Objectives:**

Despite decades of national and global strategies, persistent inequities in oral health outcomes, access, and service provision remain. Existing frameworks often fail to integrate clinical and behavioral factors with social, cultural, and political determinants. This study aimed to map and evaluate oral health frameworks using Hodges' Health Career—Care Domains‐Model (HCM), a meta‐framework that spans clinical, behavioral, sociological, and political domains. The goal was to identify conceptual gaps and opportunities for greater integration.

**Methods:**

A structured scoping review was conducted using MEDLINE, CINAHL, EBSCO, and search engine Google Scholar (1995–2025) to identify oral health‐related conceptual frameworks. Frameworks were eligible if they addressed oral health determinants, behaviors, policies, or interventions. Two reviewers independently screened records and analyzed full‐text articles. Frameworks were categorized by theoretical orientation and mapped against the four HCM domains to identify patterns of emphasis or omission.

**Results:**

Of 226 identified records, 21 frameworks met inclusion criteria. These were classified into three thematic groups: balanced (addressing all domains), clinically led (focused on clinical/behavioral aspects), and policy/public health‐focused (emphasizing sociological/political factors). Seven cross cutting themes emerged, including health promotion, systems integration, social justice, and cultural safety. While many frameworks promoted equity and policy reform, few offered implementation guidance or had been empirically validated.

**Conclusions:**

HCM proved useful for systematically comparing frameworks and revealed consistent underrepresentation of political and structural domains. It offers a practical tool for oral health professionals, educators, and policymakers developing integrated oral health models that align with equity, sustainability, and universal health coverage goals.

## 1 Introduction

Oral health research has been shaped by diverse theoretical frameworks, from behavioral theories and biopsychosocial models to socioecological and life course perspectives. Increasingly these frameworks recognize that oral health is not solely a clinical concern but is embedded in complex social, cultural, and systemic contexts. They inform the development of effective policy, service delivery, and health promotion strategies, core concerns for public health practitioners seeking to address oral health inequities.

Despite decades of global frameworks and preventive advocacy, gaps in oral health outcomes, access, and service provision persist. Oral diseases affect over 3.5 billion people worldwide and remain the most common noncommunicable conditions, with untreated dental caries in permanent teeth representing the single most prevalent health issue globally [1, 2]. Even in well‐resourced settings, with growing health literacy and community engagement, oral health continues to be described as a “neglected need” [3]. These disparities are especially evident among older adults in residential care, rural and remote populations, and Indigenous communities, who consistently face reduced access to preventive and restorative services [4, 5, 6, 7]. Oral health is therefore not solely a dental service concern but a broader public health and social equity challenge that requires coordinated, system‐wide responses addressing structural determinants. Moreover, health systems worldwide are strained by demographic change, chronic disease burdens, and social factors such as poor diet and low health literacy, further compoundng disease risks [7, 8].

These challenges persist despite global commitments such as Universal Health Coverage (UHC) and the Sustainable Development Goals (SDGs), which are international policy frameworks intended to be translated into national and regional contexts. UHC can reduce oral health inequities by expanding access to essential preventive and treatment services, while the SDGs promote multisectoral action on key determinants such as nutrition, clean water, and education (e.g., SDG 3, Target 3.8) [9, 10, 11]. Together, they articulate a plausible pathway linking global commitments to practical improvements in population oral health. However, the gap between aspirational targets and effective implementation remains wide [12, 13]. All Policies (HiAP) and intersectoral action to address the social determinants of health and equity across the prevention continuum from primordial to tertiary, are applied inconsistently [14, 15].

In response, numerous national and global oral health frameworks aim to address these challenges. While they offer strategic roadmaps for improving oral health, their impact has been variable and often limited [16], prompting calls for new conceptual models [17]. While frameworks define problems, identify intervention points, and inform policy, they frequently fall short in bridging the gap between conceptual ambition and practical implementation. Many offer limited guidance for navigating systemic barriers or adapting strategies for diverse populations.

In public health, conceptual models help simplify complexity, articulate shared values, and define professional scope, sometimes emerging from theory‐building or synthesis [18]. Unlike formal standards, which set measurable performance indicators, frameworks provide conceptual structures to identify priorities, values, and outcomes. They may function as causal or evaluative tools, highlighting key system variables, including lived experience.

Examples from general public health include WHO‐INTEGRATE, which accounts for equity, sustainability, and participation in complex decision‐making [19], and other tools that support clinical or policy decision‐making but may overlook broader structural determinants [20, 21, 22]. The FrACAS framework and VICORT checklist developed by Nguyen et al. [20] aim to enhance clinical decision‐making but do not address broader social and environmental determinants. Bracchiglione et al. [22] highlighted that many practitioners view existing frameworks as too abstract or disconnected from practical realities, while Rehfuess et al. [19] noted that general frameworks often struggle to translate conceptual ambition into actionable implementation. Collectively, these examples underscore the need for frameworks that are theoretically grounded, practically adaptable, and responsive to the contexts in which inequities emerge.

Oral health frameworks typically articulate guiding principles (e.g., equity, integration), strategic goals (e.g., reducing childhood caries), recommended actions, and mechanisms for monitoring progress. Many are developed or endorsed by The World Health Organization (WHO) or the FDI World Dental Federation, aiming to align global goals with local implementation [7, 23]. Global milestones such as the Alma‐Ata Declaration [24], WHO's Global Oral Health Strategy [24], and the FDI's Vision 2030 [25] have shaped national oral health policies, and WHO's 2023–2030 Action Plan emphasizes integration, equity, and sustainability as foundational principles [7]. Yet despite this proliferation, frameworks are frequently adopted inconsistently or applied too loosely to drive meaningful change [26]. Wolfberg [27] notes that oral health frameworks are not fixed tools but dynamic mechanisms for actionable thinking, particularly around inequities. Cabrera et al. [28] further argue that oral health promotion frameworks often overlook structural drivers of inequality, underscoring the need for models that are both theoretically grounded and practically adaptable to local contexts.

A further limitation is that most existing oral health frameworks have yet to fully embrace principles of integrated care. As Goodwin [29] argues, integrated systems must bridge care across clinical, community, and policy domains to ensure continuity and reduce fragmentation. Similarly, Fukuda‐Parr [30] observes that, global policy tools such as the SDGs often articulate aspirational goals without matching metrics or delivery mechanisms. One emerging response is the SELECT‐IT meta‐framework, which offers a four‐step process for evaluating the suitability of frameworks based on attributes like clarity, contextual responsiveness, and system integration [26]. These newer models reflect a shift toward inclusive, interdisciplinary, and systems‐aware tools, though uptake remains limited.

To address these limitations, this study applies Hodges' Health Career‐Care Domains‐Model, more commonly referred to as the Hodges' Health Care Model (HCM) [31] as a conceptual lens for analyzing the scope and structure of oral health frameworks. Conceptual models and frameworks, as described by Walker and Avant [32], play multiple roles across health disciplines, guiding the development, clarification, and evaluation of theoretical constructs. Developed by Hodges in the late 1980s [31], the HCM is a 2 × 2 matrix spanning four interdependent knowledge (care) domains: sciences (clinical/intrapersonal), interpersonal (behavioral/psychological), sociological (community/cultural), and political (policy/systems). By positioning health and social care across both individual and systemic levels, the model enables structured reflection on how determinants of health are represented [33, 34]. Building on sociological concepts of “health career” as life chances, the HCM facilitates evaluation of balance and emphasis across personal, professional, and societal dimensions of care. It has been applied across diverse fields, including mental health, aging and global health systems [35, 36, 37] and has been used to support workforce education, health promotion, intercultural communication, and policy planning [38, 39, 40, 41, 42]. While valued for its holistic perspective, some note challenges in neatly categorizing complex issues within its quadrants [41, 42], yet its explicit integration of clinical, behavioral, sociocultural, and political dimensions makes it a valuable translational praxis tool (TPT) for examining how frameworks operationalise equity and systems thinking.

Within this review, HCM is used to explore whether existing oral health frameworks adequately integrate these four domains. Specifically, the review aims to (1) systematically map and evaluate current oral health frameworks; (2) apply HCM as a meta‐framework to assess their conceptual scope and balance; and (3) identify opportunities to strengthen equity‐focused, interdisciplinary, and systems‐based approaches aligned with public health priorities and universal health coverage goals.

## 2 Methods

This review followed the scoping review methodology of Arksey and O'Malley [43], refined in subsequent guidance, to systematically identify and map oral health frameworks. To deepen the analysis, we incorporated techniques of *concept synthesis* and *concept analysis* as described by Walker and Avant [32]. This combined approach allowed us to capture the breadth of available frameworks while also interrogating their conceptual underpinnings, enabling comparative examination across models. Narrative and literary analysis were included to integrate perspectives, commentaries, and policy documents not typically captured through empirical research.

A structured search was then conducted to identify literature presenting or analyzing conceptual frameworks in oral health. Frameworks were examined for their theoretical orientation and mapped against the four care domains of HCM to identify gaps, overlaps, and underrepresented areas. This process enabled a comparative conceptual analysis of existing models in relation to an integrated, equity‐focused oral health system.

### 2.1 Literature Search and Framework Identification

A comprehensive literature search was conducted to identify existing theoretical frameworks related to oral health research. Databases searched included Medline, CINAHL, EBSCO and search engine Google Scholar, using combinations of controlled terms and free text including: frameworks, models, conceptual combined with oral health and adjacent terms including “oral health framework,” “oral health theoretical model,” “oral health conceptual framework,”, “oral health determinants,” and “oral health research models.” Searches included peer‐reviewed articles, reports, and gray literature published from 1995 to June 2025. We also included narrative reviews, perspectives, commentaries, and editorials that synthesized conceptual frameworks, theories, or policy positions that would not be captured in empirical research. Reference lists of selected studies were hand‐searched to identify additional relevant articles.

### 2.2 Inclusion and Exclusion Criteria

Inclusion criteria included reference to frameworks that explicitly aimed to conceptualize oral health determinants, behaviors, interventions, or policies. Inclusion required frameworks to conceptualize oral health determinants, behaviors, interventions, or policies, with priority given to those covering all four HCM domains; frameworks missing one or more domains were noted but excluded from final synthesis. Frameworks addressing oral health within broader health contexts were included if oral health was a primary focus. Studies from all countries were eligible, provided they met inclusion criteria related to oral health frameworks and conceptual models. *Excluded* were clinical guidelines, treatment protocols without conceptual framing, without linking to oral health outcomes, and non‐English.

### 2.3 Screening and Selection

Screening and selection were conducted using a structured spreadsheet, with duplicates and title/abstract screening managed systematically. All retrieved titles and abstracts were independently screened by two reviewers for relevance (SB and PJ). Full‐text screening was conducted to confirm eligibility. Full texts of potentially eligible articles were then reviewed to confirm inclusion. Frameworks were eligible if they explicitly or implicitly addressed all four HCM domains; 77 reports were excluded for failing to meet this criterion. Discrepancies were resolved through discussion and consensus.

### 2.4 Data Extraction and Categorization

**FIGURE 1:**
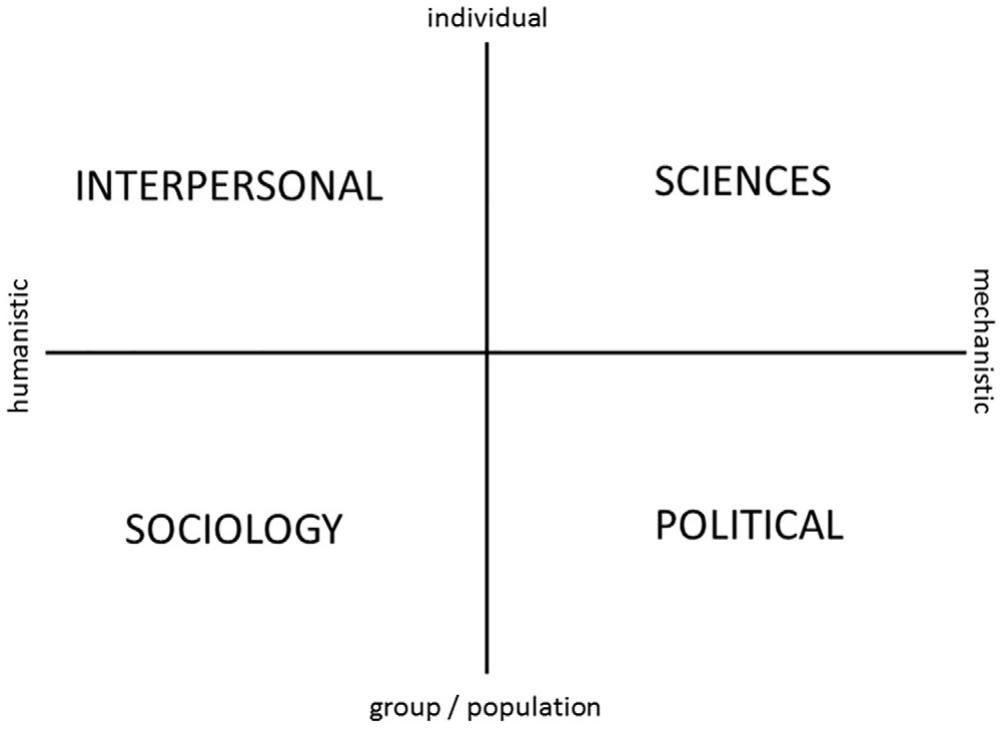
The Hodges' Health Career‐Care Domains‐Model [40] provides two axes and four domains which were used to map oral healthcare frameworks identified in the extant literature.

Data extraction items were pre‐specified in a protocol developed prior to screening. The protocol was not registered. Key characteristics of each included framework were systematically extracted. Extracted data included the authors' name, year of publication, primary focus (e.g., behavioral, social determinants, clinical), theoretical basis, and key components. To enable a structured analysis, each framework was mapped onto HCM [40] (Figure 1), which delineates four core domains influencing health.

- Clinical/Scientific (Sciences)
- Behavioral/Interpersonal (Interpersonal)
- Sociological (Social determinants)
- Political/Structural (Political)

## 3 Results

A total of 226 records were identified through database searching, comprising 121 from MEDLINE, 58 from CINAHL, and 47 from EBSCO. During the screening phase, 58 duplicate records were removed; this included overlaps between MEDLINE and CINAHL, reducing their combined records from 179 to 93, and 16 duplicates between EBSCO and MEDLINE. After de‐duplication, 168 unique records remained for screening. For eligibility assessment, full‐text articles were reviewed, resulting in 81 relevant papers from the combined MEDLINE and CINAHL search and 17 relevant papers from EBSCO, leading to a total of 98 full‐text articles assessed for eligibility.

**FIGURE 2:**
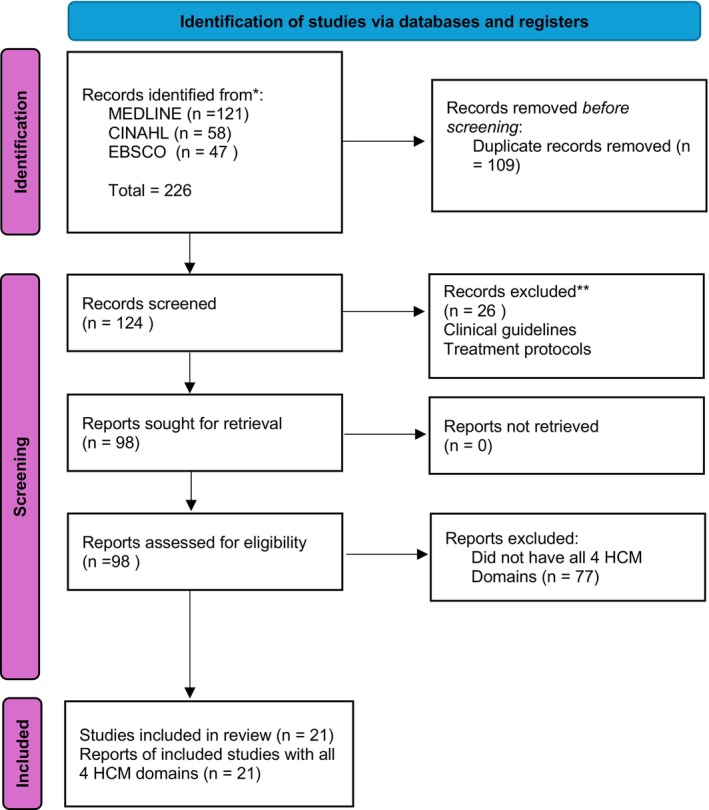
PRISMA diagram showing identification, selection and assessment of relevant literature. [Color figure can be viewed at wileyonlinelibrary.com]

The PRISMA‐ScR flow diagram was used to transparently report the identification, screening, eligibility, and inclusion of sources of evidence in this scoping review (Figure 2).

Two independent researchers assigned frameworks to one or more of these domains based on explicit and implicit content. Differences in domain assignment were resolved through consensus discussion. Frameworks referencing all four domains were identified and summarized in Table 1.

**TABLE 1:** Articles by year, author(s) and so on, mapped to Hodges' Health Career‐Care Domains‐Model across its four domains (detailed in column 9).

Year	Author(s)	Framework name or model	Study type and methodology	Health context with key features	Geographic region	Target population	Domains addressed (explicit or implicit)	HCM domains
1997	Andersen and Davidson [44]	Behavioral Model of Health Services Use (Expanded for Oral Health)	Narrative Review Conceptual framework development; synthesis of existing literature.	Oral health access and outcomes	United States (multi‐site including IHS and San Antonio)	General population, with subgroups by ethnicity and age (12–13, 35–44, 65–74 years)	External environment, healthcare system, population characteristics (predisposing, enabling, need), health behaviors, outcomes, ethnicity, age Dental care system, policy	C/B/S/P
2002	Watt [45]	Emerging Theories on Social Determinants of Health	Narrative Review Review of public health theories (life course, salutogenesis, social capital) relevant to oral health.	Oral Health Promotion	United Kingdom	General population, focus on oral disease prevention	Political (policy/health systems), Social (social determinants), Science (oral epidemiology), Individual (behavior/psychosocial)—Explicit	C/B/S/P
2005	Newton and Bower [46]	Social Determinants of Oral Health – Complex Causal Networks Framework	Narrative Review Discussion on conceptualizing complex causal networks; proposes new research approaches.	Oral Health Stress, family support, social capital	United Kingdom	General population with a focus on populations affected by social inequality	Implicitly addresses: Political (public policy), Economic (SES), Psychological (beliefs, behaviors), Sociological (social inequalities, networks), and Physical (environmental exposures) domains	C/S/P
2007	Fisher‐Owens et al. [47]	Conceptual Model of Influences on Children's Oral Health	Narrative Review Development of a conceptual model based on literature review.	Pediatric Oral Health Individual to policy‐level factors; family, SES, cultural norms, access barriers	United States	Children and their families	Political (policy/access), Social (environment/community), Science (clinical/biological), Individual (behavior/psychosocial)—Explicit	C/B/S/P
2011	Simpson [48]	Sustainable Oral Health Promotion Framework	Framework Development Proposes a staged model for implementing and sustaining oral health interventions.	Oral health promotion and intervention sustainability	USA	General population	Health promotion, sustainability, community engagement, behavior change, program implementation, evaluation Policy, training, workforce	B/P
2012	Watt and Sheiham [49]	Common Risk Factor Approach integrated into Social Determinants	Conceptual Paper Proposes integration of CRFA with social determinants of health framework.	Oral health social determinants and prevention	Global (general public health)	General population	Sociological, Individual Political (Implicit), Scientific (Implicit)	C/B/S/P
2016	Phillips and Hummel [50]	Oral Health Integration in Primary Care Framework	Framework Proposal Presents a framework for integrating oral health into primary care settings.	Integration of oral health into primary care	USA	General population, underserved populations	Oral health assessment, provider training, referral systems, health promotion, access, coordination between medical and dental care	C
2019	Lee et al. [51]	Integrating Oral Health with Public Health Systems Framework	Policy Commentary Discusses integration of oral health into public health systems using the Global Charter framework.	Global oral health integration, governance and data	Global (aligned with WHO General population, with emphasis on integrated health systems)	General population, with emphasis on integrated health systems	Public health systems, governance, policy alignment, health promotion, disease prevention, equity, multisectoral collaboration	C/P
2020	Freeman et al. [52]	Inclusion Oral Health Framework	Theoretical Framework Introduces ‘Inclusion Oral Health’ framework incorporating social exclusion and intersectionality.	Oral health equity and inclusion incorporating social exclusion and intersectionality.	United Kingdom and Canada	Underserved populations (e.g., homeless individuals, ethnic minorities)	Social determinants of health, structural barriers, policy, community engagement, intersectionality	C/B/S/P
2020	Slack‐Smith et al. [53]	Ecosocial theory and Intersectionality	Theoretical Analysis Applies ecosocial theory and intersectionality to oral health in aging populations.	Oral health in aging with focus on inequities Life course, macro drivers	Australia	Aging populations, especially marginalized groups	Sociological, Political, Interpersonal Biological processes	C/B/S/P
2021	Balasubramanian et al. [54]	Implied Analytical framework integrating supply–demand–need models and skill‐mix considerations.	Rapid Review Systematic review of workforce planning models and data sources.	Oral Health Workforce Planning	International (with UK focus)	General population (not age‐specific)	Health workforce supply, service demand, population need, skill mix, data sources	C/P
2021	Kleinman et al. [55]	Oral Health Literacy and Health Integration Framework	Conceptual framework development Theoretical synthesis and expert consensus drawing on existing literature and policy documents.	Oral and general health integration	United States	General population, with emphasis on health literacy and integration	Health literacy, organizational health literacy, oral health literacy, health systems integration, health care services and policies	C/B/P
2022	Koirala et al. [56]	Clinical Competency Framework for Basic Package of Oral Care	Qualitative Study Interviews with primary oral health providers; thematic analysis.	Primary oral health care in rural Nepal	Nepal	Primary Oral Health Providers and Clinic Assistants	Clinical competencies, professional development, performance guidance, peer accountability, job satisfaction, community impact	C/P
2022	Martin et al. [57]	Strategic Action Framework for Environmentally Sustainable Oral Healthcare	Consensus statement/policy document Delphi‐like stakeholder engagement and expert consensus building, supported by literature review.	Environmental sustainability in oral healthcare	Global	Oral healthcare providers, policymakers, industry stakeholders	Environmental impact awareness, sustainable practices, waste management, procurement, policy development, education, circular economy, stakeholder collaboration	C/P
2023	Faulks et al. [58]	International Classification of Functioning (ICF) Applied to Oral Health	Conceptual Analysis Discusses application of ICF framework to oral health measurement.	Oral health measurement and policy	International (WHO‐aligned)	General population, with emphasis on functional and social dimensions	Body functions and structures, activities and participation, environmental and personal factors, social context, disability, health systems	C/S/P
2023	Pachava [59]	Biophysical & Psychosocial Political Model integrated with the Common Risk Factor Approach (CRFA)	Editorial Advocates for integrating biophysical and psychosocial models with CRFA in oral disease prevention.	Prevention of oral diseases within the broader context of non‐communicable diseases (NCDs)	India	General population, with a focus on communities affected by oral diseases and NCDs	Biological, psychological, social, and political determinants of health	C/B/S/P
2023	Tsakos et al. [60]	Theories on oral health inequalities	Narrative Review Reviews theories and pathways related to oral health inequalities; suggests future research directions.	Oral health inequalities research	Global	Populations affected by oral health inequalities	Sociological, Political, Interpersonal (Implicit) Biological processes	B/S/P
2024	Bakri et al. [61]	Integrated Framework for Workplace Oral Health Promotion Planning and Evaluation	Framework Development Proposes a framework for workplace oral health promotion; based on literature and expert input.	Workplace oral health promotion	Australia and New Zealand	Working‐age adults in occupational settings	Health promotion theory, organizational context, individual behavior, environmental support, evaluation metrics	C/B/S
2024	Hegde et al. [62]	Co‐designed Value‐Based Healthcare (VBHC) Framework	Case Study Describes co‐design process and implementation of value‐based healthcare framework.	Public oral health service delivery	Victoria, Australia	Public dental service users and providers	Patient‐centred outcomes, co‐design, participatory action research, service redesign, value‐based care principles	B
2024	Kelly et al. [63]	Culturally Safe Oral Health Care Framework for First Nations Kidney Warriors	Participatory Research Co‐design methodology involving First Nations participants; qualitative analysis.	Oral health care for First Nations individuals with kidney disease	South Australia, Australia	Aboriginal and Torres Strait Islander individuals with kidney disease	Decolonizing methodologies, cultural safety, community engagement, participatory action research, interprofessional collaboration	B/S
2025	Lakshmikantha et al. [64]	The Pacific Islands dental research framework	Perspective Conceptual framework; descriptive and policy‐oriented methodology	Oral health research and policy development in small island nations	Pacific Islands (Fiji and broader Pacific region)	Populations of Pacific Island nations, with a focus on addressing geographic, economic, and workforce barriers in oral health	Political, Sociological (explicit) Clinical, Intrapersonal (implicit)	C/B/S/P

*Note:* Key HCM domains—clinical/scientific (C); behavioral/interpersonal (B); sociological/social determinants (S); political/structural (P).

### 3.1 Geographic Origins and Target Population

The 21 included articles covered the four domains of HCM (explicitly or implicitly) and were published between 1995 and 2025. The articles originated from the United States, United Kingdom, UK–Canada (joint), Australia (including Victoria and South Australia), New Zealand (joint AU–NZ), India, Nepal, Fiji, and several global sources, often WHO‐aligned. Most frameworks addressed the general population, often without age‐specific limitations. Several focused on subgroups such as children (aged 12–13), adults (35–44), and older adults (65–74), with particular attention to aging and marginalized populations. Children and families were a distinct focus in several studies.

A number of frameworks target underserved or vulnerable groups, including homeless individuals, ethnic minorities, and populations affected by social inequality or oral health disparities. Aboriginal and Torres Strait Islander individuals, particularly those with chronic conditions like kidney disease, are also specifically considered in relation to systemic and culturally safe care. Other frameworks addressed individuals affected by oral and non‐communicable diseases, working‐age adults, and users and providers within public dental systems. Some frameworks are oriented toward prevention, integration of oral health into broader health systems, and the social and functional aspects of oral health. While emphasis varied, all frameworks underscored the importance of equity, prevention, and context‐specific approaches tailored to population needs.

### 3.2 Framework Characteristics and Domain Mapping

The included frameworks varied in scope and domain emphasis. Some offered comprehensive models encompassing clinical, behavioral, sociological, and political factors, while others focused more narrowly, most commonly emphasizing clinical competencies or public health policy. Mapping the frameworks onto HCM care domains revealed patterns of emphasis facilitating systematic comparison across diverse frameworks. This mapping not only highlights conceptual coverage but also identifies domains where frameworks are underrepresented, providing actionable insights for the design, adaptation, and implementation of oral health strategies, a practical application of translational praxis.

Across the 21 included papers coverage across HCM care domains varied, with all frameworks addressing at least one domain. Clinical/Scientific aspects were the most frequently addressed, with 17/21 frameworks (81%) explicitly or implicitly covering clinical or scientific elements of oral health. Behavioral/Interpersonal factors, including psychosocial or interpersonal considerations, were incorporated in 13/21 frameworks (62%). Sociological or social determinants, reflecting community, cultural, or social influences, were addressed in 12/21 frameworks (57%). Political/Structural components, including policy, governance, or systemic factors, were considered in 15/21 frameworks (71%). Only 8/21 frameworks (38%) explicitly or implicitly covered all four HCM domains, highlighting that while most frameworks focused on specific domains, a smaller subset adopted a comprehensive, multi‐domain perspective.

These findings quantify the gaps in domain coverage and provide a foundation for the thematic gap discussion in Section 3.3.

### 3.3 Thematic Grouping of Oral Health Frameworks

Following domain mapping, frameworks were grouped into three thematic clusters based on their domain emphasis. Analysis revealed overarching themes reflecting the multidimensional nature of oral health determinants and interventions.

- *Balanced frameworks*—attach equal weight across all four domains
- *Clinically‐led frameworks*—emphasize clinical and behavioral domains, with weaker sociopolitical focus.
- *Public health/policy‐focused frameworks*—emphasize sociological and political domains while still referencing clinical and interpersonal elements.

Analysis revealed overarching themes reflecting the multidimensional nature of oral health determinants and interventions.

1. *Multilevel Determinants of Oral Health* **—**Several frameworks recognize oral health is shaped by interconnected individual, social, environmental, and policy factors [44, 45, 46, 47, 49]. These models emphasize addressing systemic, socioeconomic, and political influences alongside clinical factors. By bridging micro‐ to macro‐level determinants, they provide comprehensive guidance for designing holistic interventions targeting root causes.
2. *Health Promotion and Sustainability*—Frameworks focus on long‐term behavior change, prevention, and sustainability of oral health programs [45, 48, 52, 57, 59, 61]. This theme highlights upstream systemic interventions and challenges in implementing programs, particularly in resource‐constrained settings.
3. *Systems Integration and Governance*—Several authors emphasize integrating oral health within broader healthcare and public health systems [49, 50, 51, 62, 64]. Their work advocates improved service efficiency, interprofessional collaboration, and enhanced health system responsiveness. This is relevant for policymakers and workforce planners seeking coordinated, system‐level transformation in oral health care.
4. *Social Justice, Equity, and Cultural Safety*—The importance of addressing oral health disparities through a lens of social justice, equity, and cultural safety is underscored in several frameworks [45, 52, 53, 60, 63, 64]. These critically engage with issues of intersectionality and lived experience, focusing on marginalized populations and Indigenous communities to inform equitable service design and delivery.
5. *Measurement and Competency Frameworks*—The development of competency frameworks and tools for evaluating clinical function, health outcomes, and system performance has been highlighted in several studies [54, 56, 59, 62]. This theme supports evidence‐informed practice, enhances system accountability, and informs policy decisions through standardized assessment and workforce planning.
6. *Common Risk Factor and Chronic Disease Integration*—Here, oral health is positioned within the broader context of non‐communicable diseases and behavioral risk factors [49, 59]. This approach broadens the scope of interventions and strengthens the rationale for integrated funding and policy strategies.
7. *Participatory and Co‐Design Approaches* [45, 52, 53, 57, 63, 64] emphasize the involvement of communities, patients, and stakeholders in framework development and health system redesign. These participatory approaches foster greater relevance, trust, and successful implementation of oral health programs, particularly in culturally diverse and resource‐limited settings.

By identifying these thematic emphases and gaps, the mapping provides a practical foundation for targeting underrepresented domains, thereby operationalizing the insights from HCM into concrete strategies for oral health promotion and system‐level change.

## 4 Discussion

This study mapped and analyzed a range of oral health frameworks through the lens of Hodges' Health‐Care Model, which delineates four essential domains: Clinical/Scientific, Behavioral/Interpersonal, Sociological, and Political/Structural. This meta‐framework provided a valuable structured analytic tool to systematically compare diverse models, revealing their emphasis, gaps, and integration opportunities.

Our findings show that while some frameworks achieve a balanced coverage across all domains, many adopt a reductionist focus, overemphasizing clinical or behavioral factors while underrepresenting sociopolitical determinants, thereby limiting a systems‐oriented understanding of oral health. Conversely, public health and policy‐oriented frameworks often foreground structural influences but lack detailed clinical or individual behavior components. These imbalances constrain the frameworks' capacity to capture the multifaceted determinants of oral health and potentially hinder their operationalisation in diverse real‐world settings.

Across countries, oral health coverage, benefits, and delivery models vary substantially, influencing how frameworks are operationalized. For example, frameworks from the USA and UK often align with regulated public dental systems and integrated health services, whereas frameworks from Nepal, Pacific Island nations, or Australia–NZ focus on resource‐limited settings or culturally specific populations. These contextual differences help explain why certain domains, particularly the political/structural domain, were underrepresented in practice‐oriented frameworks.

Several common strengths emerged across the included frameworks. Many adopt a comprehensive, multi‐level approach, integrating individual, clinical, social, environmental, and policy‐level factors [47, 48, 49, 61, 64]. There is strong and consistent attention to equity, marginalization, and the social determinants of oral health, particularly among underserved populations [44, 52, 58, 60]. Additionally, many frameworks advocate for policy reform, regulatory alignment, and system‐level integration, reinforcing oral health as an integral part of universal health coverage and public health systems [48, 50, 51, 64]. Notably, an increasing number of models promote person‐centred, culturally sensitive, and community‐informed care [52, 56, 58, 62] while several introduce innovative concepts such as inclusion, planetary health, value‐based care, or co‐design, contributing to a broader reimagining of oral health [52, 54, 57, 62, 64].

However, these strengths are tempered by recurring limitations. Despite conceptual richness, many frameworks provide limited guidance for implementation, especially in clinical or resource‐constrained settings [56, 59, 64]. Others are resource‐intensive or require significant organizational and cultural change, posing challenges to adoption [52, 56, 58, 62]. A substantial number lack empirical validation, evaluation mechanisms, or measurable outcomes [52, 54, 59, 61]. Some frameworks are also context‐ or culture‐specific (e.g., children, rural Nepal, Pacific region), which may limit their generalizability [47, 56, 64]. Several policy or system‐level models underrepresent clinical science, behavioral mechanisms, or practice‐level strategies [48, 51, 60] highlighting the need for full integration.

HCM serves not only as a classification tool but also as a practical guide for framework development and application. It supports researchers and practitioners in identifying under‐addressed domains, promoting a holistic view of oral health. This integration is critical for advancing equity, sustainability, and effectiveness in oral health promotion and care. Its quadrant structure supports reflection on how influences are distributed and interact across clinical, behavioral, sociological, and political domains, helping identify underrepresented areas [13, 41, 42].

Building on HCM's conceptual foundation, the translational praxis tool (TPT) operationalizes reflection and action within real‐world systems [65, 66, 67]. The TPT aligns conceptual understanding with practice, facilitating iterative learning, adaptive change, and cross‐sector collaboration. Embedding HCM within a translational praxis framework strengthens its capacity to move beyond analysis toward implementation, ensuring that oral health frameworks are not only conceptually robust but also practically actionable.

Based on these findings, we recommend that future frameworks aim to strengthen coverage across all domains of HCM. In the clinical/scientific domain, this includes incorporating standardized competencies, evidence‐based care pathways, and integration into primary care. In the behavioral/interpersonal domain, health literacy initiatives, behavior‐change strategies, and co‐designed community programs are recommended. For the sociological domain, culturally safe care, targeted outreach, social inclusion initiatives, and community engagement should be prioritized. Finally, the political/structural domain can be operationalized through policy levers, workforce planning, regulatory strategies, financing models, and embedding oral health in universal health coverage. We further recommend developing evaluation mechanisms and measurable outcomes to empirically test and refine frameworks, tailoring them to country‐specific contexts while maintaining generalisability across diverse health systems. Participatory, culturally sensitive, and co‐designed approaches should also be promoted to improve uptake and relevance.

This study contributes to strengthening oral health systems by promoting both theoretically grounded and practically actionable frameworks. Applying broad frameworks within fragmented or siloed health systems is challenging; HCM provides an operational scaffold to bridge these gaps, enhance coherence, and align oral health with global health priorities such as the SDG and UHC [37, 68].

As noted, HCM is situated within practice and promotes the use of evidence‐based interventions through reflection and supervision. All clinicians hypothesize, and in therapeutic or counseling contexts, the model helps identify and weigh bio‐psycho‐socio‐political relationships. The resulting map is open rather than prescriptive, influenced by patient priorities, context of care, and agreed formulations of strengths and challenges. This flexibility allows HCM to operate as both a reflective and translational framework. At the collective or population level, its structure supports linking individual and systemic perspectives, such as connecting oral health to broader health trajectories such as cardiac and brain health or aging studies, for example [69]. In this way, HCM fosters praxis, bridging evidence, experience, and context, to inform integrated, sustainable oral health systems globally.

Ultimately, this analysis argues that oral health professionals and policymakers must be not only clinically skilled but also system‐aware, culturally responsive, and equity‐focused. Tools like HCM support this shift by fostering critical thinking, reflective practice, and situational awareness, key capabilities for advancing safe, equitable, and integrated oral health systems. Taken together, these recommendations suggest that the four domains of Hodges' model can serve not only as a heuristic framework but also as a foundational protocol for research and service development.

Beyond its value as an analytic scaffold, HCM can function as a developmental tool for assessing the maturity and capability of oral health frameworks. By mapping coverage across domains, the model highlights disparities, differentiates between health services and broader health systems, and identifies gaps against emerging standards. National dental associations and policymakers may use this approach to chart more coherent pathways for service development, benefiting from lessons learned across diverse contexts.

Overall, this mapping exercise bridges disciplinary and thematic gaps, providing a robust foundation for more comprehensive, context‐sensitive, and adaptive frameworks, encouraging interdisciplinary collaboration, and supporting development of interventions and policies better aligned with the complex realities of oral health. By highlighting strengths and weaknesses across frameworks, this study lays the groundwork for future empirical validation and the creation of adaptable, context‐sensitive models that can inform sustainable oral health systems globally.

## Conflicts of Interest

The authors declare no conflicts of interest.

## Data Availability

Data sharing not applicable to this article as no datasets were generated or analyzed during the current study.
